# Soluble Photosensitive Polyimide Precursor with Bisphenol A Framework: Synthesis and Characterization

**DOI:** 10.3390/polym17111428

**Published:** 2025-05-22

**Authors:** Bowen Zheng, Jing Li, Ning Li, Wa Li, Shuai Zhang, Haile Lei

**Affiliations:** Research Center of Laser Fusion, China Academy of Engineering Physics, Mianyang 621900, China; zhengbowen22@gscaep.ac.cn (B.Z.); jingli0218@163.com (J.L.); lncaep@163.com (N.L.); wawa44810@126.com (W.L.)

**Keywords:** photosensitive polyimide (PSPI), solubility, polymer networks (IPNs), mechanical properties, bisphenol A

## Abstract

A soluble photosensitive polyamide ester precursor (BAFPAE) was synthesized through copolymerization of 2,2-bis [4-(4-aminophenoxy)phenyl]hexafluoropropane (HFBAPP) with 4,4′-(4,4′-isopropylidenediphenoxy)bis(phthalic anhydride) (BPADA). Hydroxyethyl methacrylate (HEMA) was incorporated as a photosensitive functional group, and a transparent photosensitive polyimide film was obtained by thermal curing of the precursor film. The effects of reaction temperature and varying HEMA equivalents on the mechanical properties of the film were systematically investigated. The results indicated that the formation of polyacrylate-polyimide interpenetrating polymer networks (IPNs) was pivotal in preserving the mechanical integrity of the material. The optimized BAF-*x*-*y* exhibited a toughness of 12.69 MJ m^3^, a Young’s modulus of 2.86 GPa, an elongation at break of 21.16%, and a tensile strength of 92.68 MPa.

## 1. Introduction

Polyimide is recognized for its excellent thermal stability, chemical resistance, and mechanical performance [1,2,3,4]. It has been extensively utilized in various fields, including aerospace, electrical engineering, flexible films, high-performance plastics, adhesives, and protective coatings [5,6,7,8]. Despite their many advantages, a well-documented drawback of polyimides is their intrinsic poor solubility, which significantly hinders processability [4,9]. Therefore, a key objective of polyimide chemical modifications is to enhance their solubility in organic solvents, thereby improving processability and broadening their range of applications [4,9,10]. Numerous structural modifications have been widely studied, including the selection of diamine and dianhydride monomers, copolymerization, side-chain grafting, end-capping, and doping [10,11,12,13,14,15]. To meet the demands of rapid manufacturing and sustainable chemistry, increasing attention has been devoted to the development of soluble photosensitive polyimides (PSPIs). PSPIs maintain the intrinsic properties of polyimides while exhibiting photosensitivity. Through the synergistic effects of photocrosslinking and the polyimide network, these materials can maintain performance while improving processing and molding capabilities [16]. In recent years, substantial progress has been made in applying PSPI to microelectronic processing, advanced packaging, and additive manufacturing [17,18,19,20,21,22]. UV-curing technology employs UV light to initiate molecules with reactive double bonds, rapidly transforming them into a solid state [23,24]. UV-curing technology is characterized by its simple equipment, high efficiency, mild curing conditions, low cost, and minimal volatile organic compound (VOC) emissions, making it suitable for large-scale production [25,26,27,28]. However, soluble polyimides and their precursors are generally present as oligomers. Achieving high solubility without compromising the mechanical integrity of polyimides remains a challenge.

Interpenetrating polymer networks (IPNs) are defined as combinations of two or more polymers, each crosslinked in a network structure, in which at least one polymer is synthesized or undergoes secondary crosslinking in the presence of the other. Biphasic IPNs are generally regarded as possessing superior thermal stability and mechanical performance [29].

In this study, the photocrosslinking ability of photosensitive polyimides was used as a strategy for enhancing their mechanical properties. A highly soluble, transparent polyimide was successfully synthesized using bisphenol A dianhydride 4,4′-(4,4′-isopropylidenediphenoxy)bis(phthalic anhydride) (BPADA) and bisphenol F diamine 2,2-bis [4-(4-aminophenoxy)phenyllhexafluoropropane (HFBAPP). The integration of hydroxyethyl methacrylate (HEMA) as a photosensitive moiety imparted photocurability to the material. By modifying the chemical equivalent of HEMA, five novel photosensitive polyamide ester precursors (BAFPAEs) with distinct properties were synthesized. The effects of HEMA equivalent and imidization degree on the mechanical properties were systematically investigated by adjusting the reaction temperature. The findings demonstrated that the formation of a dual crosslinked interpenetrating network between polyimide and polyacrylate significantly enhances material toughness while preserving its mechanical strength.

## 2. Experimental

### 2.1. Materials

4,4′-(4,4′-isopropylidenediphenoxy)bis(phthalic anhydride) (BPADA), 2-hydroxyethyl methacrylate (HEMA), 1-methyl-2-pyrrolidone (NMP), tetrahydrofuran (THF), Dimethyl sulfoxide (DMSO), N,N-dimethylformamide (DMF), acetone, dichloromethane chloroform, petroleum ether (PE), ethyl acetate (EA), bis [4-(diethylamino)phenyl] ketone (DEAK), and ethyl (2,4,6-trimethylbenzoyl) phenylphosphinate (TPO-L) were procured from Shanghai Aladdin Biochemical Technology Co., Ltd. (Shanghai, China) 2,2-bis [4-(4-aminophenoxy)phenyl]hexafluoropropane (HFBAPP) was acquired from J&K Scientific (China) Co., Ltd. (Beijing, China). Methanol, ethanol, and p-toluenesulfonic acid (pTSA) were acquired from Macklin Biochemical Technology Co., Ltd. (Shanghai, China). All reagents were used without additional purification.

### 2.2. Synthesis of Bisphenol A-Bisphenol F (BAF) Photosensitive Poly(Amic Ester) (BAFPAE)

Initially, polyamide acid (PAA) was synthesized. BPADA (5.26 g, 10.1 mmol) and HFBAPP (5.18 g, 10.0 mmol) were dissolved in 100 mL of NMP within a 250 mL three-necked round-bottom flask equipped with an argon inlet and a magnetic stirrer. The solution was stirred for 4 h at room temperature. The temperature was then raised to 50 °C, and stirring was continued for 4 h. Subsequently, HEMA and pTSA were added to the PAA solution after it was heated to 70 °C. The HEMA equivalents were added at stoichiometric ratios of 40%, 60%, 80%, 100%, and 120% relative to the carboxyl groups, while the amount of pTSA was 5% of BPADA. The mixture was stirred for 12 h at 100 °C. A homogeneous brown mixture was obtained.

The resulting suspension was vacuum-filtered through a Büchner funnel, washed with deionized water (3 × 500 mL), and subsequently dried under reduced pressure at 40 °C for 12 h, yielding the BAFPAE precursor, which was a pale yellow powder. The precursors, synthesized with varying HEMA equivalents, were designated as BAFPAE-*x*, where *x* corresponds to the molar equivalents of HEMA (*x* = 0.4, 0.6, 0.8, 1.0, or 1.2).

### 2.3. Preparation of Photocurable BAFPAE-x Samples

The BAFPAE precursor was dissolved in DMF supplemented with the photoinitiators TPO-L and DEAK to formulate solutions of varying concentrations for sample fabrication. The detailed preparation parameters are provided in Table S1 (Supporting Information). For mechanical property assessments, films were fabricated by uniformly casting 2 mL of a 10 wt% BAFPAE solution onto glass substrates, followed by sequential drying at 60 °C for 2 h and 80 °C for 24 h to eliminate residual solvent. All specimens underwent UV irradiation (λ = 395 nm, irradiance: 72 mW cm^−2^) for 10 min to ensure complete photopolymerization.

### 2.4. Preparation of BAF Polyimide

The BAF precursor underwent a two-step thermal imidization process to form polyimide. Initially, the sample was heated to 100 °C at a rate of 5 °C min^−1^ in a programmable box furnace under continuous argon flow. Subsequently, a controlled temperature ramp of 0.5 °C min^−1^ was applied until the target imidization temperature was reached. The system was held isothermally for 2 h at the target temperature, followed by passive cooling to room temperature. The resulting polyimides were systematically labeled as BAF-*x*-*y*, where x denotes the molar equivalents of HEMA incorporated during synthesis (x = 0.4, 0.6, 0.8, 1.0, or 1.2) and y represents the final imidization temperature (y = 160 °C, 180 °C, 200 °C, 220 °C, or 240 °C). The cured films designated for mechanical characterization were sectioned into rectangular strips.

### 2.5. Measurements

The thickness of the cured films was measured using a digital micrometer (Model DL321025B, Deli Group Co., Ltd., Ningbo, China) with a resolution of 1 μm. Measurements were conducted at five randomly selected points on each specimen, and the average value was recorded as the representative thickness. The BAF film was mechanically ground into a fine powder using an agate mortar. The thermomechanical properties of the samples were analyzed using dynamic mechanical analysis (DMA 242 E, Shanghai Lijing Scientific Instrument Co., LTD, Shanghai, China) in tensile mode. Measurements were conducted over a temperature range of 25–250 °C at a heating rate of 10 °C min^−1^ with a frequency of 1 Hz under continuous argon purge (flow rate: 50 mL min^−1^). The glass transition temperature (Tg) was determined from the peak position of the storage modulus (E′) curve. The thermal stability of the polyimide was assessed using a thermogravimetric analyzer (TG) (HTG-4, Beijing Hengjiu Experimental Equipment Co., Ltd., Beijing, China). Measurements were carried out under continuous argon flow with a heating rate of 10 °C min^−1^ over a temperature range of 50 to 1000 °C. The solubility of BAFPAE was assessed by preparing saturated solutions in 2.0 g of various organic solvents. The tested solvents included DMF, NMP, DMSO, DCM, acetone, and ethanol. Each mixture was stirred at 150 rpm and maintained at 60 °C to reach dissolution equilibrium. The number-average molecular weight (Mn), weight-average molecular weight (Mw), and polydispersity index (PDI) were determined by a gel permeation chromatography (GPC) system (501, Surwit Technology Ink., Hangzhou, China). Hexafluoro isopropanol (HFIP) served as the eluent at a flow rate of 1.0 mL min^−1^, and the column temperature was maintained at 35.0 °C. Fourier transform infrared spectroscopy (FT-IR; Vertex 70, Nengpu Technology Co., Ltd., Tianjin, China) was employed to analyze structural changes in BAF via functional group characterization. The intensity of light irradiation was measured using an illuminometer (LS-125, Shenzhen Linshang Technology Co., Ltd., Shenzhen, China). The tensile properties of BAF films were assessed using an electronic universal testing machine (CMT, Meters Industrial Systems Co., Ltd., Shenzhen, China), with a minimum of three replicates per sample to ensure statistical validity.

### 2.6. Photosensitive Performance

The structural transformation of BAFPAE during UV-induced photopolymerization was analyzed using Fourier transform infrared spectroscopy (FT-IR). This analytical technique was employed to monitor the depletion of unsaturated moieties involved in the crosslinking process. Specifically, the conversion degree of C=C bonds in HEMA moieties was quantitatively assessed through time-resolved analysis of characteristic infrared absorption bands (806–902 cm^−1^) [30]. The progression of this photoinitiated reaction was quantified by analyzing the integrated peak area of C=C stretching vibrations, following the methodology defined in Equation (1) [31].

(1) $$\mathrm{Conversion}\;\left(\%\right)=\left(1-\frac{A_{C=C\;t}/A_{IS\;t}}{A_{C=C\;0}/A_{IS\;0}}\right)\times 100\%$$

The polymeric films were subjected to UV irradiation (395 nm wavelength, 72 mW cm^−2^ irradiance) for 600 s under controlled conditions. The FT-IR spectra of the samples were recorded at intervals of 0 s, 1 s, 2 s, 3 s, 4 s, 5 s, 10 s, 20 s, 30 s, 40 s, 50 s, 60 s, 80 s, 100 s, 120 s, 180 s, 240 s, 300 s, and 600 s. All spectral measurements were recorded in the 4000–450 cm^−1^ range at a resolution of 4 cm^−1^, with 16 scan accumulations per spectrum.

### 2.7. Imidization Degree

The imidization processes were quantified via FT-IR too. Distinct absorption bands at 1370 cm^−1^ (aromatic C-N stretching) and 1500 cm^−1^ (benzene ring vibrations) acted as diagnostic markers [19,32]. Chemical conversion was quantified by establishing relative absorbance ratios between reactive species and a reference peak, and the imidization degree (DI) was subsequently calculated using Equation (2) [33]. The subscript “sample” denotes the BAF films fabricated as described in Section 2.4, while “cured” refers to specimens subjected to complete thermal curing through 2 h of post-treatment at 240 °C.

(2) Degree of Imidization(%)=hsample(1370cm−1)hsample(1500cm−1)hcured(1370cm−1)hcured(1500cm−1)×100%

The imidization conversion rate calculated via FT-IR was treated as a relative value, whereby the sample exhibiting the highest ratio of the imide characteristic peak to the internal standard peak was assumed to be fully converted to polyimide, and the conversion rates of other samples were estimated accordingly [34,35].

Specifically, the calculation was based on the assumption that the film heated at 240 °C consisted entirely of fully imidized polyimide [36].

## 3. Result and Discussion

### 3.1. Synthesis and Characterization

**Figure 1:**
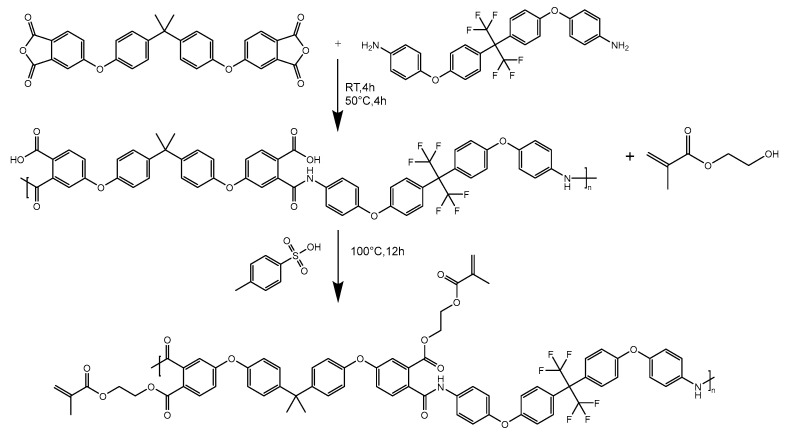
Preparation of UV-curable BAFPAE-x (HEMA).

**Figure 2:**
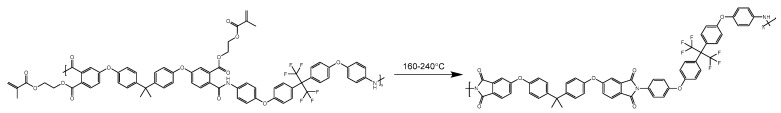
Thermal imidization process of BAF-x-y.

The BAFPAE-x precursor was synthesized via the polycondensation reaction involving bisphenol A-based BPADA and diamine HFBAPP. A schematic illustration of the synthetic route is provided in Figure 1. Polymerization was conducted under magnetic stirring and an inert argon atmosphere to enhance reaction efficiency while maintaining strictly anhydrous and oxygen-free conditions. The concentration of the reactant solution should be controlled at 10 wt% to ensure uniform stirring. Intermediates with different HEMA ratios were synthesized by heating the reaction mixture to 100 °C in the presence of p-toluenesulfonic acid as a catalyst. As depicted in Figure 2, thermal treatment of the photocrosslinked BAFPAE-x precursors yielded the final polyimide materials, designated as BAF-*x*-*y*. Here, *x* indicates the molar equivalents of HEMA incorporated during synthesis (*x* = 0.4, 0.6, 0.8, 1.0, or 1.2) and *y* specifies the final imidization temperature (*y* = 160 °C, 180 °C, 200 °C, 220 °C, or 240 °C).

**Figure 3:**
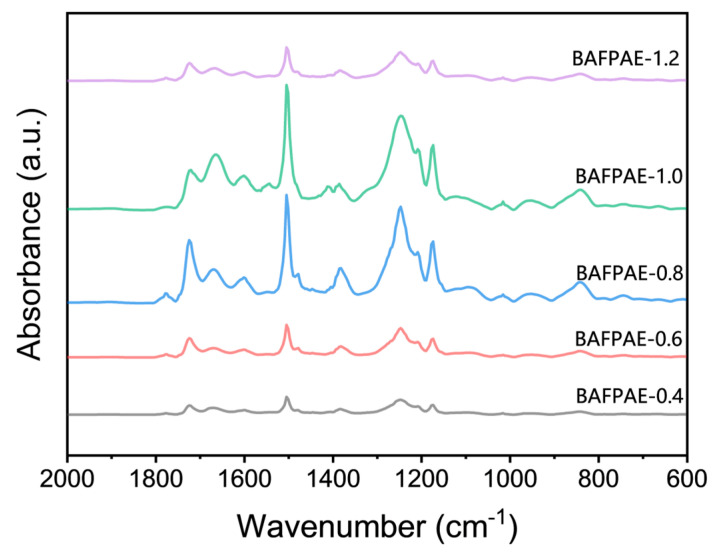
The FT-IR spectra of BAFPAE.

Fourier transform infrared (FT-IR) spectra of various BAFPAE polyamide esters were shown in Figure 3. Characteristic absorption bands were observed at 1724 cm^−1^ (C=O stretching), 1666 cm^−1^ (C=O stretching), 1550 cm^−1^ (C–N stretching), and 1500 cm^−1^ (aromatic ring skeletal vibration, C=C). The characteristic imide absorption bands typically found at 1780 cm^−1^, 1720 cm^−1^, 1370 cm^−1^, and 720 cm^−1^ were absent. Additionally, absorption peaks corresponding to HEMA moieties were identified at 1604 cm^−1^, 1410 cm^−1^, 1250 cm^−1^, and 1170 cm^−1^. Two additional absorption bands associated with HEMA, at 1320 cm^−1^ and 1095 cm^−1^, were observed exclusively in BAFPAE-0.8, BAFPAE-1.0, and BAFPAE-1.2. The absence of these two absorption peaks in the remaining samples was attributed to their comparatively lower HEMA content. Collectively, these observations confirmed that the synthesis of BAFPAE proceeded as intended, demonstrating that the untreated polymer backbone was polyamide ester rather than polyimide.

**Figure 4:**
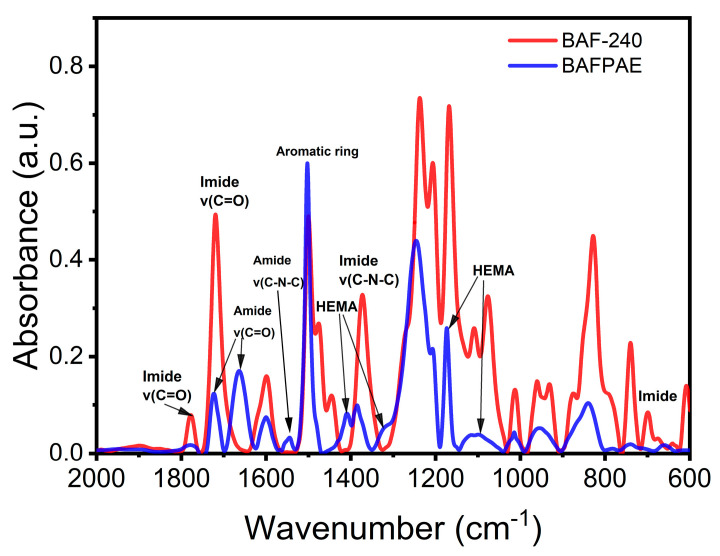
FT-IR of BAFPAE (blue) and BAF-240 (red).

Figure 4 presents the FT-IR spectra of BAFPAE and thermally treated BAF films at 240 °C. The FT-IR spectrum of BAFPAE (blue line) exhibited characteristic vibrations of the aromatic amide group at 1720 cm^−1^ (C=O stretching), 1650 cm^−1^ (C=O stretching), and 1550 cm^−1^ (C-N stretching). Additional absorption peaks corresponding to HEMA were identified at 1410 cm^−1^, 1320 cm^−1^, 1170 cm^−1^, and 1095 cm^−1^. Two absorption bands at 1600 cm^−1^ (C-H stretching) and 1500 cm^−1^ (C-C stretching) were ascribed to benzene ring vibrations [9,19,37]. Following thermal curing at 240 °C for 2 h, characteristic imide absorption bands emerged at 1780 cm^−1^, 1720 cm^−1^, 1373 cm^−1^, and 718 cm^−1^, accompanied by the disappearance of amide and HEMA-related peaks. These observations confirm that the BAF film underwent complete conversion into polyimide through the thermal treatment process.

### 3.2. Dissolution Performance

BAFPAE exhibited good solubility at room temperature in aprotic solvents such as 1-methyl-2-pyrrolidinone (NMP), N,N-dimethylformamide (DMF), methyl sulfoxide (DMSO), and tetrahydrofuran (THF). Heating (<50 °C), mechanical stirring, or ultrasonic treatment could significantly enhance dissolution efficiency. But it could not dissolve in methanol, ethanol, or petroleum ether. The maximum solubility values in different solvents were provided in Table 1. Solubility displayed a non-monotonic dependence on HEMA content, reaching a maximum at BAFPAE-0.8, with a solid content exceeding 80 wt% in DMF. Notably, BAFPAE also exhibited solubility in certain low-boiling solvents, such as acetone, DCM, and EA. It exhibited superior solubility characteristics in comparison to other polymers in its class. The bisphenol A-derived BAFPAE precursor contained benzene rings and trifluoromethyl groups, inducing significant steric hindrance. The steric hindrance exerted by the -CF_3_ and -CH_3_ groups and the flexible (-O-) segment markedly distorts the main chain, resulting in a non-coplanar and twisted conformation that effectively impedes chain stacking [38]. This structural feature disrupted the packing of molecular chains.

**Figure 5:**
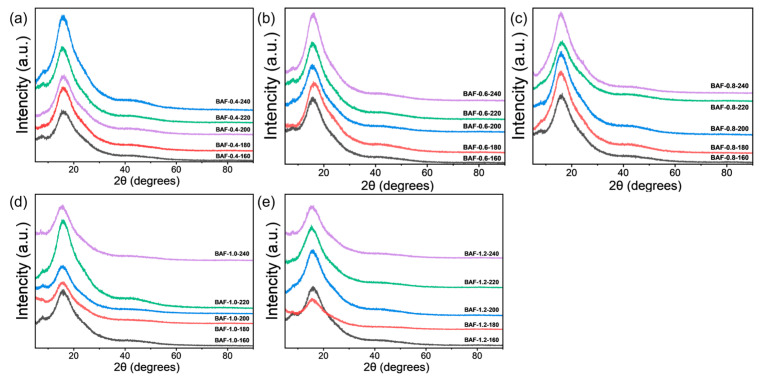
Wide-angle XRD patterns of the BAF-*x*-*y* films. (**a**) BAF-0.4-*y*; (**b**) BAF-0.6-*y*; (**c**) BAF-0.8-*y*; (**d**) BAF-1.0-*y*; (**e**) BAF-1.2-*y*.

The BAF film was analyzed by X-ray diffraction (XRD). The results revealed an amorphous halo, with no discernible signs of crystallization (Figure 5), which further elucidates the observed high solubility. The presence of a flexible propane bridge and ether linkages allowed dynamic molecular conformational adaptation during dissolution, thereby synergistically enhancing solubility.

**Figure 6:**
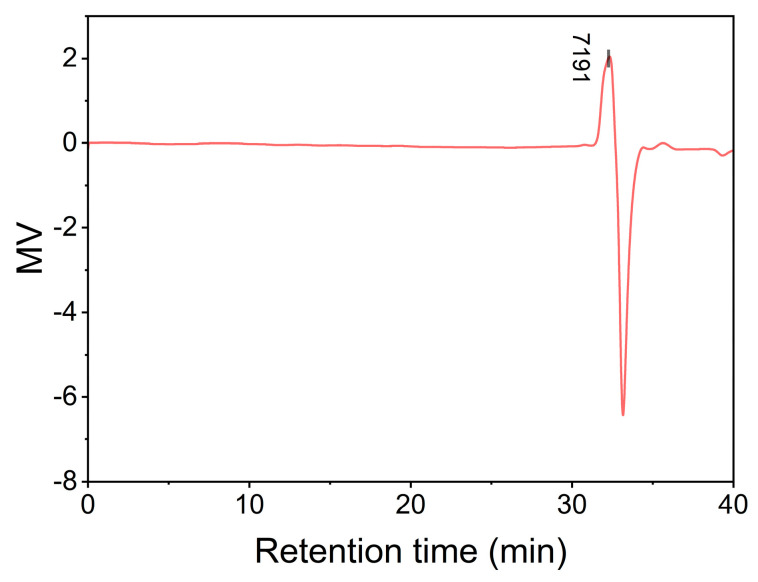
Gel permeation chromatography (GPC) results for BAFPAE.

Gel permeation chromatography (GPC) confirmed the oligomeric nature of BAFPAE (Figure 6), revealing a number-average molecular weight (Mn) of 7090, a weight-average molecular weight (Mw) of 7131, and a polydispersity index (PDI) of 1.0058. It was speculated that the degree of polymerization (*n*) was 5–6, suggesting that BAFPAE was an oligomer, which was advantageous for dissolution.

### 3.3. Photocuring Kinetics and the Degree of Imidization

**Figure 7:**
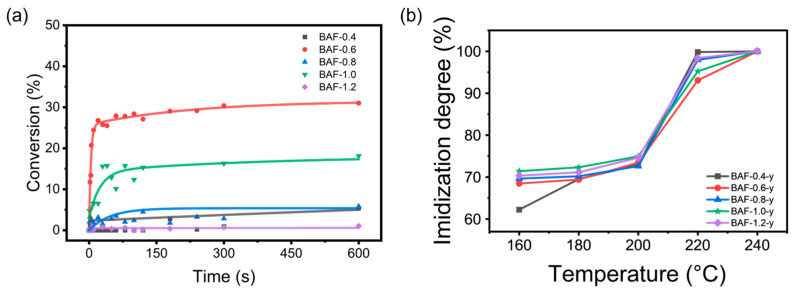
(**a**) The conversion rate of double bonds. (**b**) The imidization degree of BAF-*x*.

The double bond conversion degree was calculated using Equation (2), with results shown in Figure 7a. All BAF-x samples exhibited relatively rapid photopolymerization kinetics, typically reaching maximum double bond conversion within 20 s, where x denotes the molar equivalents of HEMA incorporated during synthesis (x = 0.4, 0.6, 0.8, 1.0, or 1.2). As the UV irradiation time increased, the conversion rate gradually rose, eventually reaching its maximum value. The maximum conversion rates for BAF-0.4 to BAF-1.2 were 5.38%, 31.02%, 1.734%, 18.10%, and 1.10%, respectively. The reaction rate and final conversion rate of BAF-1.2 were the lowest in this series, attaining only 1.1% conversion after 60 s. This indicated significant limitations in reactivity. This decrease in reactivity was attributed to the increased steric hindrance caused by higher HEMA content, which hindered double bond interactions and severely impeded completion of the reaction. Notably, BAF-0.6 exhibited significantly higher curing kinetics than other formulations, achieving a conversion rate of 11.75% within 1 s and 27.88% within 60 s. This enhanced reactivity could be attributed to the optimal balance between HEMA and carboxyl functional groups in the formulation, ensuring sufficient photosensitivity while mitigating steric hindrance effects. Furthermore, as the functionality of the acrylate groups increased beyond a certain threshold, the reduction in C=C intensity became less significant. This can be partly attributed to the polymer’s twisted, non-coplanar backbone structure, which introduces substantial steric hindrance and reduces the grafting density of HEMA monomers. Additionally, the attenuation may also result from increased functionality and viscosity, which limit the mobility and reactivity of the remaining C=C groups during polymerization [39].

As shown in Figure 7b, the degree of imidization was determined using Equation (2) following thermal treatment at 160 °C, 180 °C, 200 °C, 220 °C, and 240 °C for 2 h. At 220 °C, BAF-0.4 achieved the highest degree of imidization (99.82%), which can be attributed to its lower HEMA content, imposing minimal steric hindrance on the imidization reaction. In contrast, BAF-0.6 exhibited lower conversion efficiency than the other samples, likely due to its higher initial double bond conversion and the formation of a denser HEMA crosslinked network, both of which partially hindered polyimide formation.

### 3.4. Thermal Properties

**Figure 8:**
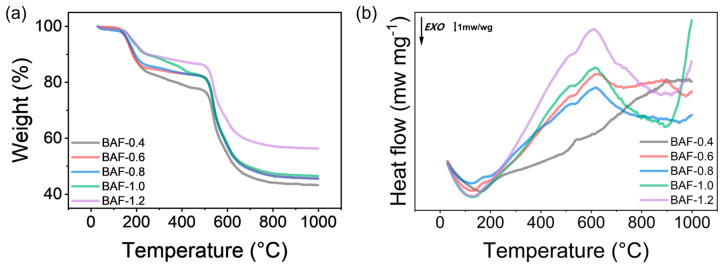
(**a**) TG curves of BAF thermal-treated at different temperatures. (**b**) DSC curves of BAFPAE precursors.

TGA and DSC were utilized to examine the thermal behavior of BAF-x precursors, as shown in Figure 8. A slight mass loss of less than 2 wt% was observed below 140 °C, likely due to the evaporation of residual solvents or moisture. The mass loss remained below 5 wt% up to 160 °C, indicating that this temperature marked the critical threshold for reaction initiation. Between 130.0 °C and 243.5 °C, BAF exhibited a weight loss of 5–10 wt%. The DSC curve in Figure 8b displayed a distinct exothermic peak within this range, signifying the imidization reactions responsible for converting BAF into polyimide. Between 232 °C and 502 °C, minimal mass loss (<3%) was recorded without detectable thermal transitions, attributed to the gradual degradation of the polyacrylate crosslinked network during polyimide formation, resulting in the slow volatilization of small molecules. The residual mass of the BAF precursor decreased with increasing HEMA content. This trend suggests a direct correlation between HEMA concentration and the release of volatile small molecules. Rapid thermal degradation of BAF was observed between 500 °C and 620 °C, accompanied by a prominent endothermic peak in the DSC curve. At 500 °C, BAF retained over 80 wt% of its initial mass, ultimately forming stable carbonaceous residues between 46% and 56% at 1000 °C. These findings underscore the exceptional thermal stability of BAF compared to conventional photosensitive resins.

**Figure 9:**
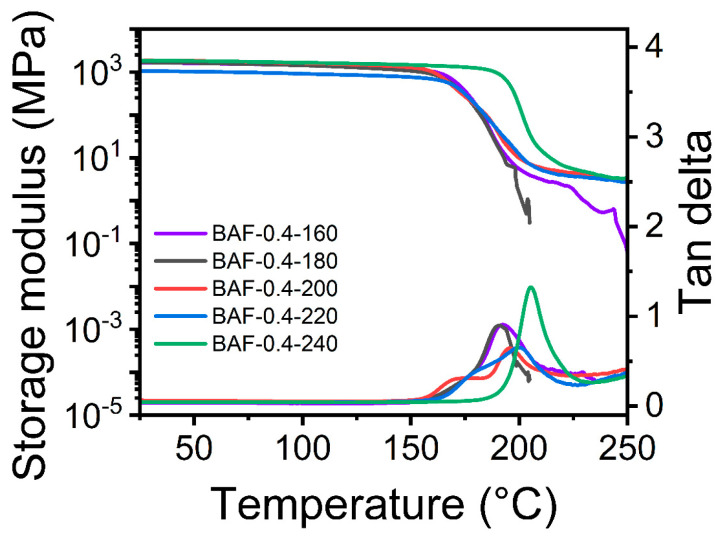
DMA curves of BAF-0.4 films cured at different temperatures.

Figure 9 presents the DMA curves of BAF-0.4 films subjected to curing at different temperatures for 2 h. Polymers with high concentrations of both polyimide and polyacrylate exhibited yielding behavior at elevated temperatures, leading to unreliable DMA measurements. Increasing the heat treatment temperature progressively enhanced the thermal properties of the materials, attributed to the superior thermal stability of the polyimide crosslinked network in comparison to the polyacrylate phase. As the thermal treatment temperature increased, the degree of imidization improved, leading to a progressive rise in the polymer’s glass transition temperature (T_g_). The sample treated at 160 °C showed an imidization degree of 62.23% and the lowest T_g_ value (191.37 °C), while the sample treated at 240 °C achieved the highest imidization conversion rate and T_g_ value (205.18 °C). These results demonstrated that the formation of a polyimide crosslinked network enhanced both the glass transition temperature and the material’s thermal stability.

### 3.5. Mechanical Properties

**Figure 10:**
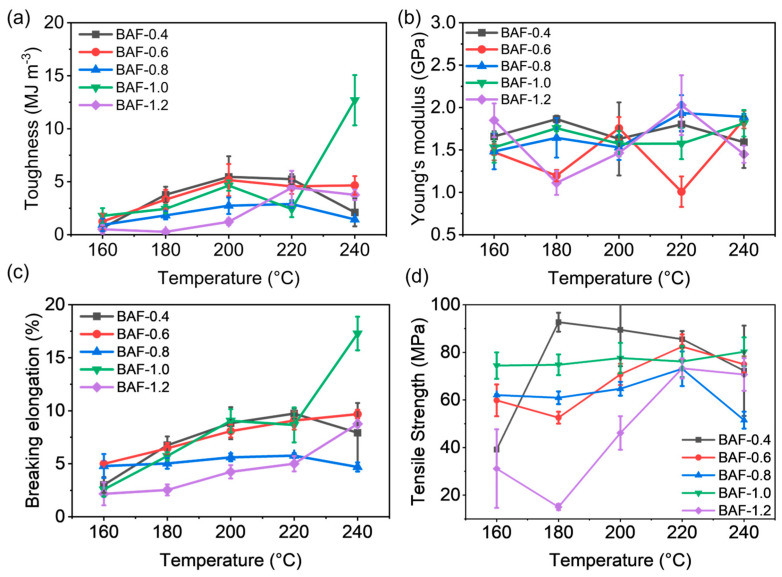
Mechanical property curves of BAF-*x*-*y*: (**a**) toughness, (**b**) Young’s modulus. (**c**) breaking elongation, and (**d**) tensile strength.

Figure 10 compares the mechanical properties of thermally cured BAF-x-y specimens subjected to different curing temperatures, where *x* denoted the molar equivalents of HEMA (*x* = 0.4, 0.6, 0.8, 1.0, or 1.2), and *y* denoted the imidization temperature (*y* = 160 °C, 180 °C, 200 °C, 220 °C, or 240 °C). As the thermal treatment temperature increased, polyimide crosslinked networks gradually formed, while polyacrylate crosslinked networks decomposed. The differing final ratios between these interpenetrating polymer networks (IPNs) resulted in varying mechanical behaviors among the samples. As the processing temperature increased, samples exhibited gradual improvements in toughness. Specifically, BAF-1.0 exhibited a significant increase in toughness of approximately 40% at 240 °C. The optimized sample (BAF-1.0-240) exhibited a toughness of 12.69 MJ m^3^, a Young’s modulus of 1.89 GPa, and an elongation at break of 21.16%. This mechanical behavior was attributed to stress yielding observed during deformation, which simultaneously enhanced both toughness and breaking elongation. The Young’s modulus (E) of BAF ranges from 1.11 GPa to 2.03 GPa, with no significant differences observed. The highest Young’s modulus of BAF-1.2-160 (2.86 GPa) was due to insufficient polyimide crosslinking, whereas the high crosslinking degree of the polyacrylate network significantly reduced toughness.

## 4. Conclusions

This study synthesized bisphenol A-type photosensitive polyimide using bisphenol A-type diamine and dianhydride monomers, incorporating HEMA at varying chemical equivalents as the photosensitive active group. The BAFPAE precursor demonstrated excellent solubility, readily dissolving in both high-boiling-point polar solvents and low-boiling-point solvents such as THF or EA at room temperature. The maximum solubility achieved was 80 wt%. The effect of imidization degree and crosslinking density was investigated using different thermal treatment temperatures. The results indicated that the crosslinked interpenetrating network of polyacrylate and polyimide collectively maintained the material’s mechanical properties. The maximum toughness of BAF-*x*-*y* was 12.69 MJ m^−3^, with a Young’s modulus of 2.86 GPa, an elongation at break of 21.16%, and a tensile strength of 92.68 MPa. The sample exhibiting the highest toughness was BAF-1.0-240, while the sample with the highest strength was BAF-0.4-180. These differences were attributed to the varying ratios of polyacrylate and polyimide. The glass transition temperature of the material was 205.18 °C, and the residual mass at 1000 °C was approximately 50%. These findings demonstrated that the material exhibited excellent thermal stability and mechanical properties, providing a valuable reference for the research and development of photosensitive polyimide materials.

## Figures and Tables

**Table 1:** Solubility of the BAFPAE precursor.

Solvent	DMF	NMP	DMSO	THF	DCM	Acetone	Ethanol	EA
BAFPAE-0.4	72 wt%	70 wt%	60 wt%	53 wt%	++	+−	−−	++
BAFPAE-0.6	76 wt%	70 wt%	60 wt%	55 wt%	++	+−	−−	++
BAFPAE-0.8	80 wt%	76 wt%	60 wt%	60 wt%	++	+−	−−	++
BAFPAE-1.0	76 wt%	75 wt%	60 wt%	55 wt%	++	+−	−−	++
BAFPAE-1.2	75 wt%	71 wt%	60 wt%	50 wt%	++	+−	−−	++

Percentage: solid content after being thoroughly dissolved; ++: soluble at room temperature; +−: partially soluble; −−: insoluble.

## Data Availability

The original contributions presented in this study are included in the article/Supplementary Materials. Further inquiries can be directed to the corresponding authors.

## Supplementary Materials

The following supporting information can be downloaded at: https://www.mdpi.com/article/10.3390/polym17111428/s1, Figure S1 ^1^H NMR spectrum of the BAFPAE (DMSO-d_6_); Figure S2 (a) FT-IR spectra of BAFPAE resin at different polymerization times; (b) The FT-IR spectra of BAF at different temperatures; Table S1 Photocurable BAFPAE resin formulations; Table S2 Gel Permeation Chromatography (GPC) Test Results for BAFPAE; Table S3 Solubility of BAFPAE precursor and other PIs in various organic solvents; Table S4 The thickness of the test film; Table S5 The mechanical properties of BAF and other PIs reported in literatures [40,41,42,43,44].

## References

[1] Dong Z., He Q., Shen D., Gong Z., Zhang D., Zhang W., Ono T., Jiang Y. (2023). Microfabrication of functional polyimide films and microstructures for flexible MEMS applications. Microsyst. Nanoeng..

[2] Mo W.J., Zhang Z.P., Rong M.Z., Zhang M.Q. (2024). Photosensitive polyimide enabled simple, reversible and environmentally friendly selective metallization on diverse substrates. Appl. Mater. Today.

[3] Ustad R.E., Chavan V.D., Kim H., Shin M.-h., Kim S.-K., Choi K.-K., Kim D.-K. (2023). Thermal, Mechanical, and Electrical Stability of Cu Films in an Integration Process with Photosensitive Polyimide (PSPI) Films. Nanomaterials.

[4] Tamai S., Yamashita W., Yamaguchi A. (1998). Preparation and properties of processable polyimides having bulky pendent ether groups. J. Polym. Sci. Part A Polym. Chem..

[5] Wu Z., He J., Yang H., Yang S. (2022). Progress in Aromatic Polyimide Films for Electronic Applications: Preparation, Structure and Properties. Polymers.

[6] Flores-Bonano S., Vargas-Martinez J., Suárez O.M., Silva-Araya W. (2019). Tortuosity Index Based on Dynamic Mechanical Properties of Polyimide Foam for Aerospace Applications. Materials.

[7] Zhou L.R., Wu G.N., Gao B., Zhou K., Liu J., Cao K.J., Zhou L.J. (2009). Study on charge transport mechanism and space charge characteristics of polyimide films. IEEE Trans. Dielectr. Electr. Insul..

[8] Sezer Hicyilmaz A., Celik Bedeloglu A. (2021). Applications of polyimide coatings: A review. SN Appl. Sci..

[9] Maya E.M., Lozano A.E., de Abajo J., de la Campa J.G. (2007). Chemical modification of copolyimides with bulky pendent groups: Effect of modification on solubility and thermal stability. Polym. Degrad. Stab..

[10] Ghosh A., Sen S.K., Banerjee S., Voit B. (2012). Solubility improvements in aromatic polyimides by macromolecular engineering. RSC Adv..

[11] Liu B., Zhou Y., Dong L., Lu Q., Xu X. (2022). Enhanced thermal conductivity in copolymerized polyimide. iScience.

[12] Kawakami Y., Yu S.-P., Abe T. (1992). Synthesis and Gas Permeability of Aromatic Polyamide and Polyimide Having Oligodimethylsiloxane in Main-Chain or in Side-Chain. Polym. J..

[13] Filippov A.P., Krasova A.S., Tarabukina E.B., Kashina A.V., Meleshko T.K., Yakimansky A.V. (2016). The effect of side chain length on hydrodynamic and conformational characteristics of polyimide-graft-polymethylmethacrylate copolymers in thermodynamically good solutions. J. Polym. Res..

[14] Kimura H., Ohtsuka K., Matsumoto A., Fukuoka H., Oishi Y. (2013). Synthesis and characterization of phenylethynylcarbonyl terminated novel thermosetting imide compound. Express Polym. Lett..

[15] Kamanina N., Toikka A., Barnash Y., Zak A., Tenne R. (2022). Influence of Surface Relief on Orientation of Nematic Liquid Crystals: Polyimide Doped with WS2 Nanotubes. Crystals.

[16] Lin Q., Zhang W., Chen L., Li Y., Ning Z., Zhang X., Xiao Y. (2024). Rigid Photosensitive Polyimide Significantly Improves the Comprehensive Performance of UV-Curing Epoxy Acrylic Resins. ACS Appl. Polym. Mater..

[17] Ahne H., Leuschner R., Rubner R. (1993). Recent advances in photosensitive polyimides. Polym. Adv. Technol..

[18] Pan D., Tian Z., He T., Ouyang Y., Chen Z., Dong F., Wang S., Liu S. Investigation of Photosensitive Polyimide with Photolithography Process and Mechanical Behavior for Wafer-Level Packaging. Proceedings of the 2024 25th International Conference on Electronic Packaging Technology (ICEPT).

[19] Fan J., Zhu T., Wu W.J., Tang S.H., Liu J.Q., Tu L.C. (2015). Low Temperature Photosensitive Polyimide Based Insulating Layer Formation for Microelectromechanical Systems Applications. J. Electron. Mater..

[20] Qin S., Jiang Y., Ji Z., Yang C., Guo Y., Zhang X., Qin H., Jia X., Wang X. (2021). Three-dimensional printing of high-performance polyimide by direct ink writing of hydrogel precursor. J. Appl. Polym. Sci..

[21] Hegde M., Meenakshisundaram V., Chartrain N., Sekhar S., Tafti D., Williams C.B., Long T.E. (2017). 3D Printing All-Aromatic Polyimides using Mask-Projection Stereolithography: Processing the Nonprocessable. Adv. Mater..

[22] Herzberger J., Meenakshisundaram V., Williams C.B., Long T.E. (2018). 3D Printing All-Aromatic Polyimides Using Stereolithographic 3D Printing of Polyamic Acid Salts. ACS Macro Lett..

[23] Shukla V., Bajpai M., Singh D.K., Singh M., Shukla R. (2004). Review of basic chemistry of UV-curing technology. Pigment Resin Technol..

[24] Sangermano M., Razza N., Crivello J.V. (2014). Cationic UV-Curing: Technology and Applications. Macromol. Mater. Eng..

[25] Jamaluddin J., Lee M.C. (2013). Properties of UV-curable solvent-free pressure sensitive adhesive. J. Adhes. Sci. Technol..

[26] Chiang T.H., Chen C.H., Wei T.-C. (2019). Characterization of UV-curable adhesives containing acrylate monomers and fluorosurfactant and their performance in dye-sensitized solar cells in long-term thermal stability tests. J. Appl. Polym. Sci..

[27] Ferracci G., Zhu M., Ibrahim M.S., Ma G., Fan T.F., Lee B.H., Cho N.-J. (2020). Photocurable Albumin Methacryloyl Hydrogels as a Versatile Platform for Tissue Engineering. ACS Appl. Bio Mater..

[28] Yu A.Z., Sahouani J.M., Webster D.C. (2018). Highly functional methacrylated bio-based resins for UV-curable coatings. Prog. Org. Coat..

[29] Frisch K.C., Klempner D., Frisch H.L. (1982). Recent advances in interpenetrating polymer networks. Polym. Eng. Sci..

[30] Herrera-González A.M., Caldera-Villalobos M., Pérez-Mondragón A.A., Cuevas-Suárez C.E., González-López J.A. (2019). Analysis of double bond conversion of photopolymerizable monomers by FTIR-ATR spectroscopy. J. Chem. Educ..

[31] Liu Y., Wu X., Sun Y., Xie W. (2018). POSS dental nanocomposite resin: Synthesis, shrinkage, double bond conversion, hardness, and resistance properties. Polymers.

[32] Li W.S., Shen Z.X., Zheng J.Z., Tang S.H. (1998). FT-IR Study of the Imidization Process of Photosensitive Polyimide PMDA/ODA. Appl. Spectrosc..

[33] Zhai Y., Yang Q., Zhu R., Gu Y. (2008). The study on imidization degree of polyamic acid in solution and ordering degree of its polyimide film. J. Mater. Sci..

[34] Kim B.-H., Park H., Park H.-Y., Moon D.-C. (2013). Degree of imidization for polyimide films investigated by evolved gas analysis-mass spectrometry. Thermochim. Acta.

[35] Makhija S.M., Pearce E.M., Kwei T.K. (1992). Kinetics of imidization of poly (amic acid) in miscible and immiscible polymer blends. J. Appl. Polym. Sci..

[36] Suzuki Y., Maekawa Y., Yoshida M., Maeyama K., Yonezawa N. (2002). Ion-beam-induced dual-tone imaging of polyimide via two-step imidization. Chem. Mater..

[37] Windrich F., Kappert E.J., Malanin M., Eichhorn K.-J., Häuβler L., Benes N.E., Voit B. (2016). In-situ imidization analysis in microscale thin films of an ester-type photosensitive polyimide for microelectronic packaging applications. Eur. Polym. J..

[38] Xia S.L., Sun Z., Yi L.F., Wang Y.H. (2013). Synthesis of soluble polyimide derived from novel naphthalene diamines for liquid crystal alignment layers and a preliminary study on the mechanism of imidization. RSC Adv..

[39] Liu P., Zhu J., Cheng L., Liu X., Liu R. (2021). Curing and properties of urethane acrylates with different functionalities under electron-beam and ultraviolet irradiation. Prog. Org. Coat..

[40] Wu Y., Ding C., Yu J., Huang P. (2023). Synthesis and Characterization of Semi-Aliphatic Polyimide Films with Excellent Comprehensive Performance. Polym. Sci. Ser. B.

[41] Liu C., Zhao X., Li Y. (2013). New autophotosensitive semiaromatic hyperbranched polyimides with excellent thermal stabilities and low birefringences. High Perform. Polym..

[42] Liu Z., Zhang S., Yuan J. (2021). New developments in intrinsic black photosensitive polyimide for advanced display applications. Mater. Today Chem..

[43] She Y.K., Wang S.X., Liao Q. (2024). Transparent and highly organosoluble aromatic polyimides with twisted backbone and bulky side substituents for flexible substrate materials. J. Polym. Sci..

[44] Zhao W.J., Tong Y.Z., Zeng P.P., Zhou Y.S., Cao X.W., Wu W. (2024). Comparative study of intrachain versus interchain cross-linking on the mechanical, thermal and dielectric properties of low-k polyimide. Chin. J. Polym. Sci..

