# Supplementary material for: Soluble Photosensitive Polyimide Precursor with Bisphenol A Framework: Synthesis and Characterization
Source: Polymers (Basel). 2025 May 22;17(11):1428. doi: 10.3390/polym17111428 (PMC12157256; doi:10.3390/polym17111428)
Supplement: Supplementary file 1 [file polymers-17-01428-s001.zip › polymers-3606913-supplementary.pdf]

Supporting Information

Bo-wen Zheng, Jing Li, Ning Li, Wa Li, Shuai Zhang* and Haile Lei*

Research Center of Laser Fusion, China Academy of Engineering Physics, Mianyang 621900, PR China

*Corresponding author.

E-mail address: zhangshuai_scu@126.com (S. Zhang)

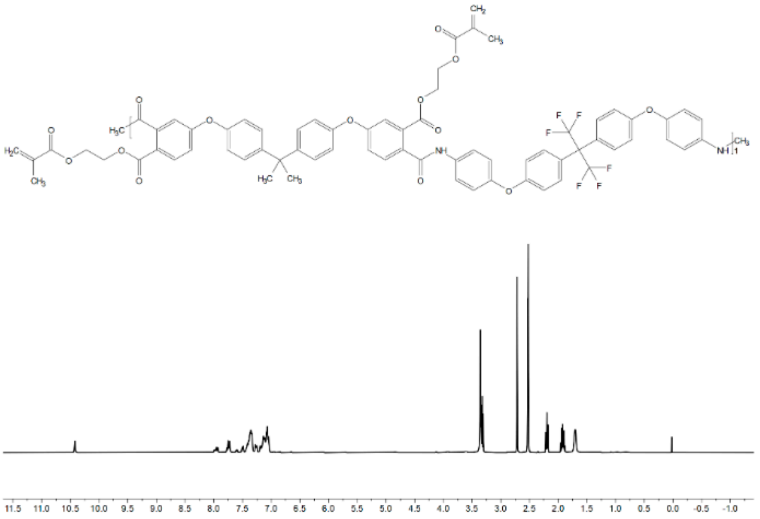

Figure S1 ^{1}H NMR spectrum of the BAFPAE (DMSO-d_6).

Figure S1 presented the ^{1}H NMR spectrum of the pc-PAE resin. The peaks located at 2.50 ppm and 3.33 ppm were assigned to solvent (DMSO-d_6) and moisture, respectively. The absorption peaks observed at 10.5 ppm and 7.52-8.26 ppm were attributed to the protons of the amide groups (-CONH-) and benzene rings in the polymer backbone, respectively. The peaks observed at 1.72 ppm, 1.89-1.96 ppm, and 6.90-7.52 ppm were assigned to the protons of the $-CH_3$, $-OCH_2-$, and $=CH_2$ groups, respectively, confirming the expected chemical structure of pc-PAE.

Table S1 Photocurable BAFPAE resin formulations.

Materials	BAFPAE -1	BAFPAE -2
BAFPAE precursor	3	20
DMF	97	80

The photoinitiator TPO-L and DEAK was added at a concentration of 1wt% relative to the BAFPAE.

The thin film utilized for Fourier transform infrared spectroscopy (FT-IR) analysis was prepared from BAFPAE-1, whereas the thin film employed for mechanical performance evaluation was prepared using BAFPAE-2. After the BAFPAE precursor solution was prepared as specified in Table S1, photoinitiators TPO-L and DEAK (1 wt% relative to precursor mass) were added. The resulting mixture was heated to 40 °C, thoroughly stirred, and subsequently cast onto a clean glass slide. Afterward, the film was thermally cured under specific conditions to yield specimens suitable for subsequent characterization. It was widely accepted that the photopolymerization of HEMA primarily proceeded via the carbon–carbon double bonds (C=C) within the acrylate groups. The reduction in C=C intensity at the sample surface was less pronounced than that in the subsurface region, suggesting a lower degree of polymerization at the surface compared to the subsurface. This phenomenon may have been attributed to the surface's exposure to atmospheric conditions, where residual oxygen acted as an inhibitor of the polymerization reaction. To mitigate this undesirable effect, two photoinitiators (DEAK and TPO-L) were incorporated into the resin formulation [1].

Table S2 Gel Permeation Chromatography (GPC) Test Results for BAFPAE

Distribution Name	Mn (Daltons)	Mw (Daltons)	MP (Daltons)	Mz (Daltons)	Mz+1 (Daltons)	Polydispersity
PAE	7090	7131	7191	7172	7213	1.0058

Table S3 Solubility of BAFPAE precursor and other PIs in various organic solvents.

**Polyimide**	**Solvent**							
	DMF	NMP	NVP	DMSO	THF	TCM	Acetone	Ethanol
**BAFPAE (this work)**	80%	76%	60%	60%	++	+-	--	++
**PAE (previous work)**	55%	40%	26%	25%	50%	--	--	--
**PI-0** [2]	++	++	○	++	○	-	++	○
**PAAS** [3]	○	⩾30%	○	○	○	○	○	○

Percentage: solid content after thoroughly dissolved; ++: soluble at room temperature; +: soluble on heating; --: insoluble; ○: not reported.

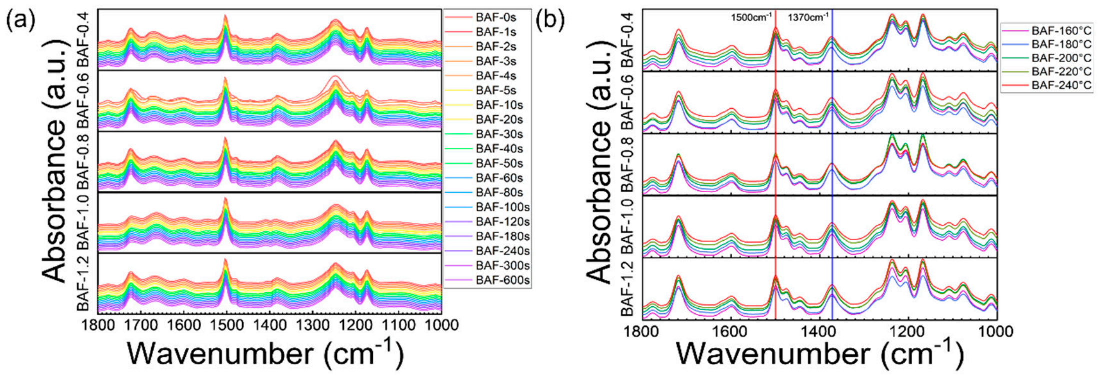

Figure S2 (a) FT-IR spectra of BAFPAE resin at different polymerization times;(b) The FT-IR spectra of BAF at different temperatures.

Table S4 The thickness of the test film

Samples	Thickness (mm)		
BAF-0.4-160	0.0559	0.0533	0.0540
BAF-0.6-160	0.0673	0.0686	0.0635
BAF-0.8-160	0.0780	0.0656	0.1041
BAF-1.0-160	0.0426	0.0498	0.0637
BAF-1.2-160	0.0271	0.0268	0.0247
BAF-0.4-180	0.0798	0.0733	0.0762
BAF-0.6-180	0.0619	0.0382	0.0596
BAF-0.8-180	0.0729	0.0643	0.0974
BAF-1.0-180	0.0831	0.0483	0.0410
BAF-1.2-180	0.0268	0.0754	0.0323
BAF-0.4-200	0.0736	0.0644	0.0706
BAF-0.6-200	0.0627	0.0609	0.0626
BAF-0.8-200	0.0983	0.0780	0.0836
BAF-1.0-200	0.0492	0.0526	0.0770
BAF-1.2-200	0.0537	0.0562	0.0575
BAF-0.4-220	0.0845	0.0910	0.0877
BAF-0.6-220	0.0664	0.0498	0.0667

BAF-0.8-220	0.0747	0.1167	0.0813
BAF-1.0-220	0.0457	0.0364	0.0479
BAF-1.2-220	0.0517	0.0456	0.0380
BAF-0.4-240	0.0807	0.0794	0.1089
BAF-0.6-240	0.0941	0.0682	0.0516
BAF-0.8-240	0.0658	0.0764	0.0701
BAF-1.0-240	0.0491	0.0385	0.0651
BAF-1.2-240	0.0637	0.0515	0.0536

When processing the mechanical testing results,the size differences among all samples have been excluded.

Table S5 The mechanical properties of BAF and other PIs reported in literatures.

Polyimide	Tensile strength (MPa)	Young's modulus (GPa)
BAF (this work)	92.68	2.86
HBPDA-ODA [4]	90.3	2.69
PHBPI-1b [5]	84	3.22
BPSPI-50 % [6]	62	2.3
PAPNT-OPDA [7]	51.3	2.1
NEAT PI [8]	52.2	○

Reference

1. Liu, P., Zhu, J., Cheng, L., Liu, X., Liu, R. Curing and properties of urethane acrylates with different functionalities under electron-beam and ultraviolet irradiation. Progress in Organic Coatings 2021, 106252.
2. Maya, E. M.; Lozano, A. E.; De Abajo, J.; De la Campa, J. G. Chemical modification of copolyimides with bulky pendent groups: effect of modification on solubility and thermal stability.*Polymer Degradation and Stability* **2007** *92*(12), 2294-2299.
3. Qin S.; Jiang Y.; Ji Z. Three-dimensional printing of high-performance polyimide by direct ink writing of hydrogel precursor. *Journal of Applied Polymer Science* **2021**, 138(27): 50636.
4. Wu, Y.; Ding. C.; Yu, J.; Huang, P.; Synthesis and Characterization of Semi-Aliphatic Polyimide

Films with Excellent Comprehensive Performance. *Polymer Science, Series B* **2023**; 65(2), 120-128.

5. Liu C; Zhao X; Li Y; New autophotosensitive semiaromatic hyperbranched polyimides with excellent thermal stabilities and low birefringences. *High Performance Polymers* **2013**; 25(3), 301-311.
6. Liu Z; Zhang S; Yuan J; New developments in intrinsic black photosensitive polyimide for advanced display applications.*Materials Today Chemistry* **2021**; 42, 102346.
7. She Y. K; Wang S. X; Liao Q; Transparent and highly organosoluble aromatic polyimides with twisted backbone and bulky side substituents for flexible substrate materials. *Journal of Polymer Science* **2024**; 62(6), 1061-1073.
8. Zhao W. J; Tong Y. Z; Zeng P. P; Zhou Y. S; Cao X. W; & Wu W.. Comparative study of intrachain versus interchain cross-linking on the mechanical, thermal and dielectric properties of low-k polyimide. *Chinese Journal of Polymer Science* **2024**, *42*(11), 1824-1834.
